# Two New Steroidal Monoglycosides, Anthenosides A_1_ and A_2_, and Revision of the Structure of Known Anthenoside A with Unusual Monosaccharide Residue from the Starfish *Anthenea aspera*

**DOI:** 10.3390/molecules23051077

**Published:** 2018-05-03

**Authors:** Timofey V. Malyarenko, Natalia V. Ivanchina, Olesya S. Malyarenko, Anatoly I. Kalinovsky, Pavel S. Dmitrenok, Evgeny V. Evtushenko, Chau Van Minh, Alla A. Kicha

**Affiliations:** 1 G.B. Elyakov Pacific Institute of Bioorganic Chemistry, Far Eastern Branch of the Russian Academy of Sciences, Pr. 100-let Vladivostoku 159, Vladivostok 690022, Russia; ivanchina@piboc.dvo.ru (N.V.I.); malyarenko.os@gmail.com (O.S.M.); kaaniv@piboc.dvo.ru (A.I.K.); paveldmt@piboc.dvo.ru (P.S.D.); evt@piboc.dvo.ru (E.V.E.); kicha@piboc.dvo.ru (A.A.K.); 2 School of Natural Sciences, Far Eastern Federal University, Sukhanova Str. 8, Vladivostok 690000, Russia; 3 Institute of Marine Biochemistry, Vietnam Academy of Science and Technology, 18 Hoang Quoc Viet, Caugiay, Hanoi, Viet Nam; cvminh@vast.vn

**Keywords:** steroidal glycosides, NMR spectra, amino sugars, starfish, *Anthenea aspera*, cytotoxicity, soft agar assay

## Abstract

Two new polyhydroxysteroidal glycosides, anthenosides A_1_ (**1**) and A_2_ (**2**), and one previously known steroidal glycoside anthenoside A (**3**) were isolated from extract of the tropical starfish *Anthenea aspera*. Structures of **1**–**3** were determined by analysis of the spectroscopic data as well as chemical transformations. As a result, the structure of anthenoside A has been revised and the structures of **1** and **2** were established. Glycosides **1**–**3** contain a 2-acetamido-2-deoxy-4-*O*-methyl-β-d-glucopyranosyl residue, found in the starfish steroidal glycosides for the first time. All the isolated compounds slightly inhibited cell viability of human cancer T-47D cells and did not show cytotoxic effects against RPMI-7951 cells. Glycoside **1** slightly inhibited colony formation of human cancer RPMI-7951 cells by 16% while compound **2** decreased the number of colonies of T-47D cells by 40%.

## 1. Introduction

As characteristic secondary metabolites of starfish (Echinodermata, Asteroidea) steroidal glycosides may be divided into three major classes: glycosides of polyhydroxysteroids, cyclic glycosides, and asterosaponins [1,2,3,4,5,6,7]. Starfish steroidal glycosides have been reported to show a wide spectrum of biological activities [1,2,3,4,5,6,7]. Polyhydroxysteroidal glycosides from starfish have highly oxygenated steroidal aglycons and in most cases monosaccharides of these compounds are represented by β-d-xylopyranosyl and α-l-arabinofuranosyl residues or their methylated or sulfated derivatives. β-d-Glucopyranosyl and β-d-galactofuranosyl residues and their derivatives also occur sometimes in the starfish steroidal glycosides. A series of steroidal glycosides containing β-d-galactofuranosyl, 6-*O*-methyl-β-d-galactofuranosyl, 3-*O*-methyl-β-d-galactofuranosyl, 3-*O*-methyl-β-d-glucopyranosyl and 4-*O*-methyl-β-d-glucopyranosyl residues was isolated from the starfishes of the genus *Anthenea* [8,9,10]. On the other hand, amino sugars are atypical monosaccharide residues in starfish steroidal glycosides but often found in sponges steroidal glycosides [11,12]. Several amino sugars, in particular β-*N*-acetyl- and β-*N*-formylgalactosamines, were found in oligosaccharide chains of gangliosides isolated from the starfish of genus *Evasterias* [13,14].

It has been previously reported, that some polyhydroxysteroidal glycosides from the starfish *Anthenea chinensis* exhibited significant activity against promotion of tubulin polymerization in vitro, inhibiting the proliferation of human leukemia K-562, hepatoma BEL-7402, and spongioblastoma U87MG cell lines [8]. Recently, we have established the structures of ten related new polyhydroxysteroidal glycosides, anthenosides L–U, from the starfish *Anthenea aspera*. Some of these glycosides showed hemolytic activity and anthenoside O exhibited reduction of ROS level, when RAW 264.7 murine macrophages cells were co-stimulated with pro-inflammatory endotoxin, lipopolysaccharide (LPS) from *E. coli* [9].

Herein, we report the results of the structural elucidation of two new polyhydroxysteroidal glycosides (**1**, **2**) and the revision of the structure of the known steroidal glycoside **3**, all containing amino sugars. Compounds **1**–**3** were isolated from the ethanolic extract of the starfish *A. aspera* collected at Tu Long Bay near Khuan Lan Island in the Vietnamese water. Moreover, we examined the effect of these compounds on the colony formation of melanoma and breast cancer cells using soft agar assay.

## 2. Results and Discussion

### 2.1. Structure Elucidation of Compounds ***1**–**3***

**Figure 1:**
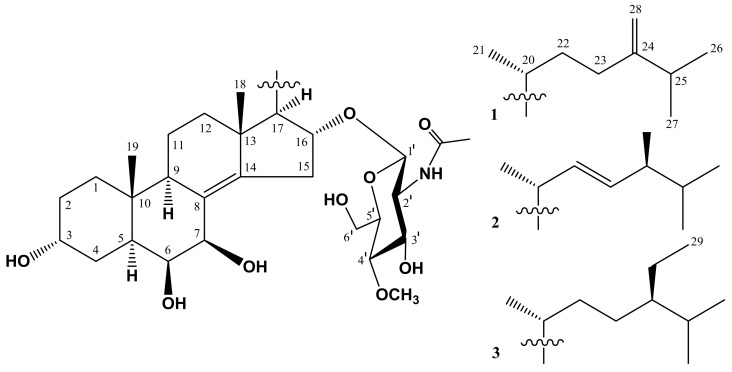
The structures of compounds **1**–**3** isolated from *A. aspera.*

The concentrated ethanol extract of *A. aspera* was subjected to sequential separation by chromatography on columns with Polychrom-1 and silica gel followed by HPLC on semipreparative Diasorb-130-C16T, and analytical Discovery C_18_ and Diasorb-130 Si gel columns to obtain two new steroidal monoglycosides, named as anthenosides A_1_ (**1**) and A_2_ (**2**), and one previously known steroidal glycoside anthenoside A (**3**) (Figure 1).

**Figure 2:**
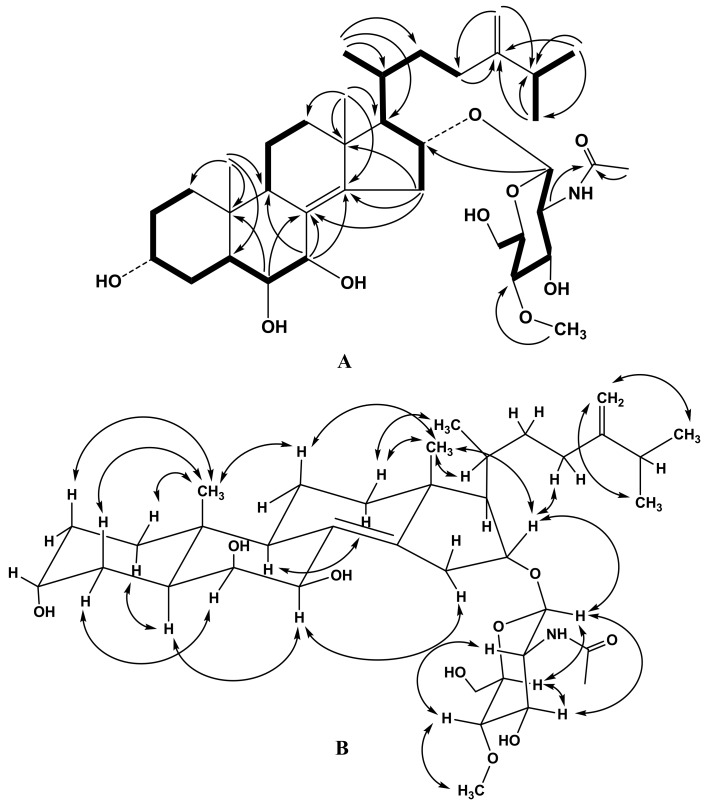
(**A**) ^1^H-^1^H COSY and key HMBC correlations for compound **1**. (**B**) Key ROESY correlations for compound **1**.

The molecular formula of compound **1** was determined to be C_37_H_61_NO_9_ from the [M + Na]^+^ sodium adduct ion peak at *m/z* 686.4238 in the (+)HRESIMS (Figure S1). The ^1^H- and ^13^C-NMR spectroscopic data belonging to the tetracyclic moiety of the aglycon of **1** showed the resonances of protons and carbons of two angular methyl groups CH_3_-18 and CH_3_-19 (*δ*_H_ 0.92 s, 0.83 s; *δ*_C_ 20.2, 15.4), a 8(14) double bond (*δ*_C_ 128.0, 147.4), three oxygenated methines HC-3 (*δ*_H_ 4.08 m; *δ*_C_ 67.4), HC-6 (*δ*_H_ 3.53 m; *δ*_C_ 77.3), HC-7 [*δ*_H_ 4.26 d (*J* = 2.7); *δ*_C_ 72.2], and one oxygenated HC-16 [*δ*_H_ 4.55 td (*J* = 9.1, 5.1); *δ*_C_ 79.8], the latter carbon being linked to a monosaccharide residue through an ether function (Table 1, Figures S2 and S3). Based on these data the tetracyclic moiety of the aglycon of **1** was determined as Δ^8(14)^-3α,6β,7β,16α-tetrahydroxysteroidal moiety glycosylated at C-16 position which is characteristic of starfish steroidal glycosides of the genus *Anthenea* [8,9,10]. The ^1^H-^1^H COSY and HSQC correlations attributable to steroidal nucleus revealed the corresponding sequences of protons from C-1 to C-7, C-9 to C-12 through C-11, and C-15 to C-17 (Figure 2A, Figures S4 and S5). Key HMBC cross-peaks, such as H-6/C-8, C-10; H-7/C-8, C-9, C-14; H-15/C-8, C-13, C-14; H_3_-18/C-12, C-13, C-14, C-17; H_3_-19/С-1, С-5, С-9, С-10 confirmed the overall structure of the steroidal moiety of **1** (Figure 2A, Figure S6). The key ROESY cross-peaks showed the common 5α/9α/10β/13β stereochemistry of the steroidal nucleus and 6β,7β,16α-configurations of oxygenated substituents in **1** (Figure 2B, Figure S7). The NMR spectroscopic data of the aglycon side chain indicated the existence of three methyl groups CH_3_-21 [*δ*_H_ 1.02 d (*J* = 6.9); *δ*_C_ 21.0], CH_3_-26 [*δ*_H_ 1.04 d (*J* = 6.8); *δ*_C_ 22.4], CH_3_-27 [*δ*_H_ 1.04 d (*J* = 6.8); *δ*_C_ 22.6] and a 24(28) double bond [*δ*_H_ 4.74 br. d (*J* = 1.0), 4.73 br. s; *δ*_C_ 158.3, 106.7) (Figures S2 and S3). The proton sequences from H-17 to H-21 and H-23 and from H-26 to H-27 through H-25, correlated with the corresponding carbon atoms of the side chain of **1**, were assigned using the ^1^H-^1^H COSY and HSQC experiments (Figure 2A, Figures S4 and S5). The HMBC correlations H_3_-21/C-17, C-20, C-22; H_3_-26/C-24, C-25, C-27; H_3_-27/C-24, C-25, C-26; H_2_-28/C-23, C-25 and ROESY correlations H-28/H-26, H-27 supported the total structure of the Δ^24(28)^-24-methyl-cholestane side chain (Figure 2A,B, Figures S6 and S7) previously found in anthenosides F, G and S [8,9]. The 20*R*-configuration was assumed on the basis of ROESY correlations of H_3_-18/H-20, H_3_-21; H_β_-16/H-22, and H_3_-21/H_β_-12. Thus, the structure of steroidal aglycon of glycoside **1** was determined to be (20*R*)-24-methyl-5α-cholesta-8(14),24(28)-diene-3α,6β,7β,16α-tetraol.

The ^1^H-NMR spectrum of **1** exhibited one resonance in the deshielded region due to the anomeric proton of a monosaccharide unit at *δ*_H_ 4.51, that correlated in the HSQC experiment with a carbon signal at *δ*_C_ 100.9 as well as one resonance due to an *O*-methyl group at *δ*_H_ 3.55, that correlated in the HSQC experiment with a carbon signal at *δ*_C_ 60.9 (Table 1, Figures S2 and S3). The (+)ESIMS/MS of the ion [M + Na]^+^ at *m/z* 686 and the (–)ESIMS/MS of the ion [M − H]^−^ at *m/z* 662 contained the fragment ion peaks at *m/z* 258 [С_9_H_17_NO_6_ + Na]^+^ and 234 [С_9_H_16_NO_6_]^−^, respectively, which together with the NMR spectroscopic data confirmed the presence of a 2-acetamido-2-deoxy-*O*-methyl-hexose unit in **1**. The chemical shifts and coupling constants of H-1′ − H-6′ of this monosaccharide unit were determined by irradiation of anomeric proton in a 1D TOCSY experiment. The ^1^H-^1^H COSY and HSQC correlations attributable to monosaccharide unit revealed the corresponding sequences of protons at C-1′ − C-6′ (Figure 2A, Figures S4 and S5). The key HMBC correlations H-1′/C-2′, C-3′, C-5′; H-2′/C-1′, C-3′, C-4′, CH_3_C=O; H-4′/O-CH_3_; O-CH_3_/C-4′; and ROESY correlations H-1′/H-3′, H-5′; H-3′/H-5′; H-2′/H-4′; H-4′/O-CH_3_ supported the total structure of the 4-*O*-methyl-2-acetamido-2-deoxy-glucopyranosyl residue (Figure 2A,B, Figures S6 and S7). The coupling constant (8.3 Hz) of the anomeric proton corresponded to a β-configuration of the glycoside bond. The D-series of monosaccharide unit was expected by analogy with co-occurring glycoside **3**. The attachment of the 4-*O*-methyl-2-acetamido-2-deoxy-β-d-glucopyranosyl residue to C-16 of steroidal aglycon was determined by the HMBC and ROESY spectra, where cross-peaks between H-1′ of 4-OMe-GlcNAc and C-16/H-16 of aglycon, respectively, were observed (Figure 2A, B, Figures S6 and S7). Based on these results, the structure of anthenoside A_1_ was determined as (20*R*)-16-*O*-(2-acetamido-2-deoxy-4-*O*-methyl-β-d-glucopyranosyl)-24-methyl-5α-cholesta-8(14),24(28)-diene-3α,6β,7β,16α-tetraol (**1**).

The molecular formula of compound **2** was determined to be C_37_H_61_NO_9_, the same as in the glycoside **1**, from the [M + Na]^+^ sodium adduct ion peak at *m/z* 686.423941 in the (+)HRESIMS (Figure S8). The thorough comparison of the ^1^H- and ^13^C-NMR data (Figures S9 and S10) of compound **2** with those of **1** showed that they differed from each other only in the signals of their steroidal side chains (Table 1). The NMR spectra of the side chain of **2** indicated the existence of four methyl groups CH_3_-21 [*δ*_H_ 1.08 d (*J* = 7.1); *δ*_C_ 24.4], CH_3_-26 [*δ*_H_ 0.87 d (*J* = 6.8); *δ*_C_ 20.6], CH_3_-27 [*δ*_H_ 0.85 d (*J* = 6.8); *δ*_C_ 20.2], CH_3_-28 [*δ*_H_ 0.96 d (*J* = 6.8); *δ*_C_ 18.3], and a 22(23) double bond [*δ*_H_ 5.77 ddd (*J* = 15.4, 9.2, 1.0), 5.24 dd (*J* = 15.4, 8.0); *δ*_C_ 135.9, 134.9] (Table 1, Figures S9 and S10). The proton sequences from H-17 to H-28 of the side chain of glycoside **2**, correlating with the corresponding carbon atoms, were assigned using ^1^H-^1^H COSY and HSQC experiments (Figures S11 and S12). The HMBC and ROESY correlations supported the total structure of the unoxidized Δ^22^-24-methyl-cholestane side chain previously found in anthenosides M–P [9] (Figures S13 and S14). The 20*R*-configuration was assumed on the basis of ROESY correlations of H_3_-18/H-20, H_3_-21 and H-22/H-16 H_3_-21/H_β_-12. The *S*-configuration at C-24 was suggested based on the chemical shift of C-28 at *δ*_C_ 18.3 (*δ*_C_ 18.1 for 24*S* and 17.4 for 24*R* configurations in the ^13^C-NMR spectra of mycalosides A and K having similar side chains [15,16]). The *trans*-configuration of a 22(23)-double bond followed from a *J*_22,23_ of 15.4 Hz (Table 1, Figure S9). Thus, the structure of anthenoside A_2_ was established as (20*R*,22*E*,24*S*)-16-*O*-(2-acetamido-2-deoxy-4-*O*-methyl-β-d-glucopyranosyl)-24-methyl-5α-cholesta-8(14),22(23)-diene-3α,6β,7β,16α-tetraol (**2**).

^1^H- and ^13^C-NMR, and MS spectral data of steroidal glycoside **3** coincided with those reported earlier for anthenoside A [17]. Previously the structure of anthenoside A was established as (20*R,*24*R*)-16-*O*-(4-*O*-methyl-2-acetamido-2-deoxy-β-d-galactopyranosyl)-24-ethyl-5α-cholest-8(14)-ene-3β,6β,7β,16α-tetraol [17]. However, we examined a number of inaccurate conclusions previously made about some structural features of anthenoside A.

The configuration of the OH-group at C-3 in the steroidal nucleus of anthenoside A was erroneously established as β-OH based on the values of chemical shifts of H-3 and C-3 at *δ*_H_ 4.10 and *δ*_C_ 67.4 in the ^1^H- and ^13^C-NMR spectra [17]. It was previously reported that the values of chemical shifts of H-3 and C-3 in a 3β-OH configuration were *δ*_H_ 3.60 and *δ*_C_ 72.5 as in anthenosides D and F [8]. The values of chemical shifts of H-3 and C-3 of the 3α-OH configuration are in the range of *δ*_H_ 4.06–4.09 and *δ*_C_ 67.3–67.5 as in anthenosides E, G, L, M, S1 and other steroidal glycosides isolated from the starfishes of the genus *Anthenea* [8,9,10].

Moreover, the width of the multiplet of H-3 of 12.5 Hz corresponded well to a 3α-OH configuration while the width of the multiplet of H-3 at the 3β-OH configuration was more than 30 Hz [8,9,10]. Thereby, the structure of the steroidal moiety of **3** should be revised as 5α-cholest-8(14)-ene-3α,6β,7β,16α-tetraol.

In addition, we revised the structure of the monosaccharide residue in anthenoside A, previously established as 2-acetamido-2-deoxy-4-*O*-methyl-β-d-galactopyranose [17]. We have analyzed the values of the coupling constants of H’-3, H’-4 and H’-5 of the monosaccharide residue of glycoside **3** in the ^1^H-NMR spectrum. The value of the common coupling constant between H’-3 and H’-4 is 8.8 Hz and the value of the common coupling constant between H’-4 and H’-5 is 9.8 Hz. It is known that the values of the coupling constants of vicinal protons in the cyclohexane ring (chair conformation) occupying the axial-axial position are more than 5 Hz (usually 8–10 Hz) such as in glucopyranosyl, xylopyranosyl and quinovopyranosyl residues [1,2,3,4,5,6,7]. The values of the coupling constants of vicinal protons in the cyclohexane ring (chair conformation) occupying the axial-equatorial position are less than 5 Hz (usually 2–4 Hz), as for example, in galactopyranosyl and fucopyranosyl residues [1,2,3,4,5,6,7]. Moreover, a cross-peak between H’-2 and H’-4 of **3** was observed in the ROESY spectrum. The cross-peak between H’-2 and H’-4 in the ROESY or NOESY spectra is observed only in glucopyranosyl, xylopyranosyl and quinovopyranosyl residues, but not in galactopyranosyl and fucopyranosyl residues [8,9,10]. Acid hydrolysis of glycoside **3** with 2 M TFA was carried out to determine a stereochemical series of its monosaccharide moiety. Alcoholysis of the obtained monosaccharide by (*R*)-(−)-2-octanol followed by acetylation, GC analysis, and comparison with the corresponding derivatives of standard monosaccharides allowed us to establish the D-configuration of the monosaccharide unit. Hence, based on the obtained data the structure of the monosaccharide residue of anthenoside A (**3**) was established as 2-acetamido-2-deoxy-4-*O*-methyl-β-d-glucopyranose.

In addition, the values of coupling constants of H_3_-26/H-25 (6.7 Hz) and H_3_-27/H-25 (6.8 Hz) of **3** were refined. These are characteristic of methyl groups of aglycon side chain in starfish polar steroidal compounds [1,2,3,4,5,6,7]. Previously, it was erroneously reported that the values of these coupling constants of anthenoside A were 11.5 Hz and 12.0 Hz, respectively [17].

Thus, the structure of the previously known anthenoside A (**3**) was corrected to (20*R*,24*R*)-16-*O*-(2-acetamido-2-deoxy-4-*O*-methyl-β-d-glucopyranose)-24-ethyl-5α-cholesta-8(14)-ene-3α,6β,7β,16α-tetraol.

### 2.2. Biological Evaluation

#### 2.2.1. The Effect of Compounds **1**–**3** on Cancer Cells’ Viability

In the present work, the cytotoxic activities of compounds **1**–**3** against human melanoma RPMI-7951 and breast cancer T-47D cell lines were tested by MTS assay. Compounds **1**, **2** and **3** slightly inhibited cell viability of T-47D cells, with IC_50_ of 158, 133, and 139 µM, respectively, but they did not have cytotoxic effects against RPMI-7951 cells at concentrations up to 150 μM (data not shown).

#### 2.2.2. Effect of Compounds **1**–**3** on Colony Formation

**Figure 3:**
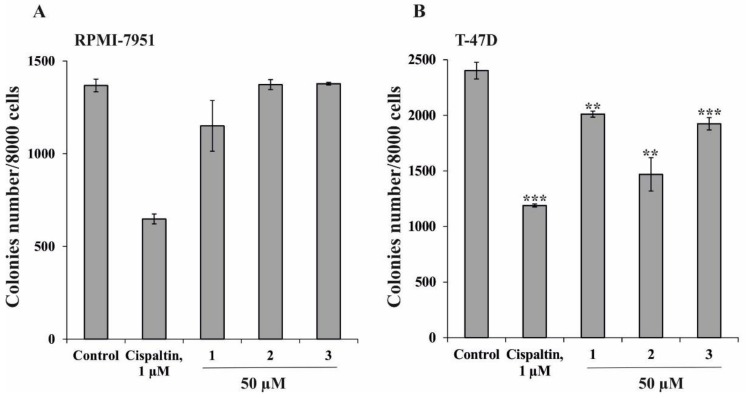
Effect of compounds **1**–**3** on colony formation of human melanoma RPMI-7951 (**A**) and breast cancer T-47D cells (**B**). The number of colonies were scored using a microscope «Motic AE 20» with the aid of the ImageJ software (*n* = 9 for control and each compound, *n*—quantity of photos). The magnification of representative photos of colonies is ×10. The asterisks indicate a significant decrease in colony formation in cells treated with compounds compared with the non-treated cells (control) (** *p* < 0.01, *** *p* < 0.001).

Next we examined the effect of **1**–**3** (50 µM) on the colony formation of human melanoma RPMI-7951 and breast cancer T-47D cells using soft agar assay. In this work cisplatin was used as a positive control, because it is a well known drug for cancer therapy [18]. It was shown that only compound **1** but not **2** and **3** slightly inhibited colony formation of RPMI-7951 cancer cells by 16% (Figure 3A). On the other hand, compounds **1** and **3** slightly decreased the number of colonies of T-47D cells by 20% and 22%, respectively (Figure 3B). Compound **2** was the most effective in this experiment with a percentage of inhibition (40%) comparable to that of cisplatin (45%). Our data are in accordance with the results obtained earlier, showing that their growth inhibitory effect of starfish steroidal glycosides is more prominent on human breast cancer cells and less prominent on melanoma cells [19,20].

## 3. Experimental Section

### 3.1. General Procedures

Optical rotations were determined on a PerkinElmer 343 polarimeter (Waltham, MA, USA). The ^1^H- and ^13^C-NMR spectra were recorded on Bruker Avance III 700 spectrometer (Bruker, Karlsruhe, Germany) at 700.13 and 176.04 MHz, respectively, chemical shifts were referenced to the corresponding residual solvent signal (*δ*_H_ 3.30/*δ*_C_ 49.0 for CD_3_OD). The HRESIMS spectra were recorded on a Bruker Impact II Q-TOF mass spectrometer (Bruker, Bremen, Germany); the samples were dissolved in MeOH (c 0.001 mg/mL). HPLC separations were carried out on an Agilent 1100 Series chromatograph (Agilent Technologies, Santa Clara, CA, USA) equipped with a differential refractometer; Diasorb-130-C16T (11 µm, 250 × 16 mm, Biochemmack, Moscow, Russia), Discovery C_18_ (5 µm, 250 × 4 mm, Supelco, North Harrison, PA, USA) and Diasorb-130 Si gel (6 µm, 250 × 4.6 mm, Biochemmack, Moscow, Russia) columns were used. GC analysis was performed on an Agilent 6850 Series chromatograph (Agilent Technologies, Santa Clara, CA, USA), equipped with a capillary column HP-5 MS (30 m × 0.25 mm) over the temperature range 100–270 °C at 5 °C/min with the carrier gas He (1.7 mL/min); the temperatures of the injector and the detector were 250 and 270 °C, respectively. Low-pressure liquid column chromatography was carried out with Polychrom-1 (powdered Teflon, 0.25–0.50 mm; Biolar, Olaine, Latvia) and Si gel KSK (50–160 µm, Sorbpolimer, Krasnodar, Russia). Sorbfil Si gel plates (4.5 × 6.0 cm, 5–17 µm, Sorbpolimer, Krasnodar, Russia) were used for thin-layer chromatography.

### 3.2. Animal Material

Specimens of *Anthenea aspera* Döderlein, 1915 (order Valvatida, family Oreasteridae) were collected at a depth of 3–20 m by hand using scuba at Tu Long Bay near Khuan Lan Island in the Vietnamese water in May 2007, during the 34th scientific cruise of the research vessel Akademik Oparin. Species identification was carried out by Dr. T.I. Antokhina (Severtsov Institute of Ecology and Evolution, RAS, Moscow, Russia). A voucher specimen [no. 034-142] is on deposit at the marine specimen collection of the G.B. Elyakov Pacific Institute of Bioorganic Chemistry of the FEB RAS, Vladivostok, Russia.

### 3.3. Extraction and Isolation

The fresh animals of *A. aspera* (1.3 kg, crude weight) were chopped into small pieces and extracted thrice with EtOH. The H_2_O/EtOH layer was evaporated, and the residue was dissolved in H_2_O (1.0 L). The H_2_O-soluble material was passed through a Polychrom-1 column (7 × 26.5 cm), eluted with distilled H_2_O (4.0 L) until a negative chloride ion reaction was obtained, and then eluted with EtOH (3.5 L). The combined EtOH eluate was evaporated to give a reddish residue (7.0 g). This material was chromatographed over a Si gel column (6 × 18.5 cm) using CHCl_3_/EtOH (stepwise gradient, 6:1–EtOH, *v*/*v*) to yield eight fractions, 1–8, which were then analyzed by TLC in the eluent system BuOH/EtOH/H_2_O (4:1:2, *v*/*v*/*v*). Fractions 1–6 mainly contained the polyhydroxysteroids and related glycosides and admixtures of pigments and concomitant lipids. HPLC separation of fraction 5 (212 mg) on a Diasorb-130-C16T column (2.5 mL/min) with EtOH/H_2_O (75:25, *v*/*v*) as an eluent system yielded pure **3** (8.5 mg, R_t_ 47.8 min) and sub-fractions 5.6 (4.0 mg) and 5.7 (13.0 mg), which were additionally submitted to a purification on Discovery C_18_ analytical column (1.0 mL/min) with EtOH/H_2_O (70:30, *v*/*v*) as an eluent system to give pure **1** (3.5 mg, R_t_ 15.5 min) and sub-fraction 5.72 (8.5 mg). HPLC separation of sub-fraction 5.72 on a Diasorb-130 Si gel analytical column (1.0 mL/min) with EtOAc/EtOH (30:1, *v*/*v*) as an eluent system yielded pure **2** (2.5 mg, R_t_ 54.7 min).

### 3.4. Compound Characterization Data

*Anthenoside A_1_* [(20*R*)-16-*O*-(2-acetamido-2-deoxy-4-*O*-methyl-β-d-glucopyranosyl)-24-methyl-5α-cholesta-8(14),24(28)-diene-3α,6β,7β,16α-tetraol] (**1**): Amorphous powder; [α]${}_{D}^{25}$: –50.6 (*c* 0.1, MeOH); IR (KBr) *ν*_max_ 3550, 3478, 3417, 2962, 2924, 2852, 1737, 1637, 1618, 1467, 1262, 1098, 1032, 801 cm^−1^; HRESIMS *m/z* 686.4238 [M + Na]^+^ (calcd for C_37_H_61_NO_9_, 686.4239); ESIMS/MS of the ion at *m*/*z* 686: *m*/*z* 258 [С_9_H_17_NO_6_ + Na]^+^; ESIMS/MS of the ion at *m*/*z* 662: *m*/*z* 234 [С_9_H_16_NO_6_]^−^; ^1^H- and ^13^C-NMR data, see Table 1.

*Anthenoside A_2_* [(20*R*,22*E*,24*S*)-16-*O*-(2-acetamido-2-deoxy-4-*O*-methyl-β-d-glucopyranosyl)-24-methyl-5α-cholesta-8(14),22(23)-diene-3α,6β,7β,16α-tetraol] (**2**): Amorphous powder; [α]${}_{D}^{25}$: −53.1 (*c* 0.15, MeOH); IR (KBr) *ν*_max_ 3550, 3478, 3417, 2962, 2924, 2858, 1735, 1637, 1618, 1467, 1262, 1098, 1033, 801 cm^−1^; HRESIMS *m/z* 686.4239 [M + Na]^+^ (calcd. for C_37_H_61_NO_9_, 686.4241); ESIMS/MS of the ion at *m*/*z* 686: *m*/*z* 258 [С_9_H_17_NO_6_ + Na]^+^; ESIMS/MS of the ion at *m*/*z* 662: *m*/*z* 234 [С_9_H_16_NO_6_]^−^; ^1^H- and ^13^C-NMR data, see Table 1.

*Anthenoside A* [(20*R*,24*R*)-16-*O*-(2-acetamido-2-deoxy-4-*O*-methyl-β-d-glucopyranosyl)-24-ethyl-5α-cholest-8(14)-ene-3α,6β,7β,16α-tetraol] (**3**): Amorphous powder; [α]_D_: –48.3 (*c* 0.35, MeOH). ^1^H-NMR (CD_3_OD, 700.13 MHz): *δ*_H_ 4.55 (1H, td, *J* = 9.1, 5.1 Hz, H-16), 4.51 (1H, d, *J* = 8.3 Hz, H-1′), 4.26 (1H, d, *J* = 2.7 Hz, H-7), 4.08 (1H, m, H-3), 3.83 (1H, dd, *J* = 11.5, 2.1 Hz, H-6′a), 3.67 (1H, dd, *J* = 11.5, 5.4 Hz, H-6′b), 3.62 (1H, dd, *J* = 10.4, 8.8 Hz, H-3′), 3.55 (3H, s, 4′-OCH_3_), 3.53 (1H, m, H-6), 3.52 (1H, dd, *J* = 10.4, 8.3 Hz, H-2′), 3.24 (1H, ddd, *J* = 9.8, 5.4, 2.1 Hz, H-5′), 3.05 (1H, dd, *J* = 9.8, 8.8 Hz, H-4′), 2.90 (1H, ddd, *J* = 17.0, 8.8, 3.0 Hz, H-15), 2.34 (1H, ddd, *J* = 17.0, 5.2, 2.1 Hz, H’-15), 2.24 (1H, m, H-9), 2.17 (1H, dt, *J* = 13.0, 2.7 Hz, H-5), 1.99 (3H, s, CH_3_-CO), 1.96 (1H, m, H-4β), 1.82 (1H, dt, *J* = 12.4, 3.6 Hz, H-12β), 1.76 (1H, m, H-25), 1.67 (1H, m, H-22), 1.65 (1H, m, H-11α), 1.62 (2H, m, H-2), 1.61 (1H, m, H-20), 1.59 (1H, m, H-23), 1.53 (2H, m, H-1α and H-11β), 1.45 (1H, dd, *J* = 9.1, 5.5 Hz, H-17), 1.37 (1H, m, H-4α), 1.36 (1H, m, H-28), 1.35 (1H, m, H’-22), 1.32 (1H, m, H’-28), 1.31 (1H, m, H-1β), 1.27 (1H, m, H-12α), 1.06 (1H, m, H’-23), 1.04 (1H, m, H-24), 1.00 (3H, d, *J* = 6.9 Hz, H_3_-21), 0.92 (3H, s, H_3_-18), 0.89 (3H, t, *J* = 7.4 Hz, H_3_-28), 0.88 (3H, d, *J* = 6.7 Hz, H_3_-26), 0.85 (3H, d, *J* = 6.7 Hz, H_3_-27), 0.83 (3H, s, H_3_-19); ^13^C-NMR (CD_3_OD, 176.04 MHz): *δ*_C_ 173.8 (CH_3_-C=O), 147.4 (C-14), 128.0 (C-8), 100.9 (C-1′), 82.0 (C-4′), 79.7 (C-16), 77.3 (C-6 and C-5′), 76.2 (C-3′), 72.2 (C-7), 67.4 (C-3), 63.0 (C-6′), 62.7 (C-17), 60.9 (4′-OCH_3_), 58.4 (C-2′), 47.5 (C-24), 45.6 (C-9), 45.0 (C-13), 38.6 (C-10), 37.7 (C-5), 37.2 (C-12), 34.5 (C-1), 34.3 (C-15), 33.8 (C-20), 33.4 (C-4), 33.3 (C-22), 30.2 (C-25), 29.5 (C-2 and C-23), 24.1 (C-28), 23.2 (CH_3_-CO), 21.2 (C-21), 20.2 (C-18), 20.0 (C-26), 19.5 (C-11 and C-27), 15.4 (C-19), 12.7 (C-29); (+)-HRESIMS: *m/z* 702.4549 [M + Na]^+^ (calcd for C_37_H_61_NO_9_, 702.4546); ESIMS/MS of the ion at *m*/*z* 702: *m*/*z* 258 [С_9_H_17_NO_6_ + Na]^+^.

### 3.5. Acid Hydrolysis and Sugar Analysis

Acid hydrolysis of **3** (1.5 mg) was carried out in a solution of 2 M trifluoroacetic acid (TFA, Sigma-Aldrich Corporation, St. Louis, MO, USA) (0.75 mL) in a sealed vial on a H_2_O bath at 100 °C for 2 h. The H_2_O layer was washed with CHCl_3_ (3 × 1 mL) and concentrated in vacuo. One drop of concentrated TFA and 0.5 mL of (*R*)-(–)-2-octanol (Sigma-Aldrich Corporation, St. Louis, MO, USA) were added to the sugar mixture, and the sealed vial was heated in a glycerol bath at 130 °C for 6 h. The solution was evaporated in vacuo and treated with a mixture of pyridine-acetic anhydride (1:1, 0.5 mL) for 24 h at room temperature. The acetylated 2-octylglycosides were analyzed by GC using the corresponding authentic samples prepared by the same procedure. The following peaks for monosaccharides were detected in the hydrolyzate of **3**: 2-acetamido-2-deoxy-4-*O*-methyl-d-glucopyranose (R_t_ = 16.21 and 20.08 min). The retention times of the authentic samples were as follows: 2-acetamido-2-deoxy-4-*O*-methyl-d-glucopyranose (R_t_ = 16.20 and 20.09 min), and 2-acetamido-2-deoxy-4-*O*-methyl-β-l-glucopyranose (R_t_ = 16.07 and 20.00 min).

The sample of 2-acetamido-2-deoxy-4-*O*-methyl-α-d-glucopyranose was synthesized as described previously [21].

### 3.6. Bioactivity Assay

#### 3.6.1. Reagents and Antibodies

MTS (3-(4,5-dimethylthiazol-2-yl)-5-(3-carboxymethoxyphenyl)-2H-tetrazolium) assay kit was purchased from “Promega” (Madison, WI, USA).

The Basal Medium Eagle (BME), Minimum Essential medium (MEM), Roswell Park Memorial Institute medium (RPMI 1640), phosphate buffered saline (PBS), l-glutamine, gentamicin solution, trypsin, fetal bovine serum (FBS), sodium hydrocarbonate (NaHCO_3_), and agar were purchased from “Sigma” and “Gibco” (Mendota Heights, MN, USA). All other common chemicals, solvents and reagents were of the highest grade available from various commercial sources.

#### 3.6.2. Cell Lines and Culture Conditions

Human melanoma RPMI-7951 cells (ATCC # HTB-66^TM^), and human breast cancer cells T-47D (ATCC # HTB-133^TM^) were obtained from the American Type Culture Collection (ATCC, Manassas, VA, USA).

Human melanoma RPMI-7951, and breast cancer T-47D cells were cultured in MEM/10% FBS and RPMI-1640/10% FBS media, respectively. The cell cultures were maintained at 37 °C in humidified atmosphere containing 5% CO_2_. The cells were grown for 3–4 days and after reaching 90% of confluence were harvested by exposure to 0.25% Trypsin-EDTA solution and then passed into new T-75 tissue culture flasks.

#### 3.6.3. MTS Assay

To determine cytotoxicity, RPMI-7951 and T-47D cells (1 × 10^4^/200 µL) were seeded in 96-well plates for 24 h at 37 °C in a 5% CO_2_ incubator. The attached cells were fed with fresh medium containing various concentrations of compounds **1**–**3** from (0–150 µM) for an additional 24 h. After, the cytotoxicity of **1**–**3** was measured using an MTS assay kit according to the manufacturer’s instructions. All the experiments were performed in triplicate, and the mean absorbance values were calculated. IC_50_ is the concentration of compounds that caused a 50% reduction of cell viability of human cancer cells after 48 h of treatment.

#### 3.6.4. Soft Agar Assay

RPMI-7951 and T-47D cells (8 × 10^3^ cells/mL) were treated with/without cisplatin (1 μM) and compounds **1**–**3** (50 µM) in 1 mL of 0.3% Basal Medium Eagle (BME) agar containing 10% FBS, 2 mM l-glutamine, and 25 µg/mL gentamicin. The cultures were maintained at 37 °C in a 5% CO_2_ incubator for 14 days, and the cell’s colonies were scored using a microscope «Motic AE 20» (China) and the Motic Image Plus computer program.

#### 3.6.5. Statistical Analysis

All assays were performed at least in triplicate. The results are expressed as the mean ± standard deviation (SD). A Student’s *t*-test was used to evaluate the data with the following significance levels: ** *p* < 0.01, *** *p* < 0.001.

## 4. Conclusions

Two new polyhydroxysteroidal glycosides, anthenosides A_1_ (**1**) and A_2_ (**2**), and one previously known steroidal glycoside anthenoside A (**3**) were isolated from the alcoholic extract of the tropical starfish *Anthenea aspera*. Chemical structures of **1** and **2** were elucidated by extensive NMR and ESIMS techniques as well as by chemical transformations. The structure of the known glycoside **3** was revised by NMR spectroscopy and GC analysis of acetylated 2-octylglycosides. Glycosides **1**–**3** contain a new monosaccharide residue, 2-acetamido-2-deoxy-4-*O*-methyl-β-d-glucopyranose, never found before in the starfish polar steroidal compounds. It is worth noting that amino sugars are atypical monosaccharide residues in starfish steroidal glycosides but often found in the steroidal glycosides of sponges. Steroidal glycosides **1**–**3** slightly inhibited cell viability of breast cancer T-47D cells but they did not have cytotoxic effects against human melanoma RPMI-7951 cells. It was shown that only compound **1** slightly inhibited colony formation of RPMI-7951 cancer cells by 16%. On the other hand, the new glycoside **2** decreased the number of colonies of T-47D cells by 40%.

## Figures and Tables

**Table 1:** ^1^H (700.13 MHz) and ^13^C (176.04 MHz) NMR chemical shifts of **1** and **2** in CD_3_OD, at 30 °C, *δ* in ppm, *J* values in Hz.

Position		1			2	
	DEPT	*δ*_H_	*δ*_C_	DEPT	*δ*_H_	*δ*_C_
1β 1α	CH_2_	1.30, m 1.53, m	34.5	CH_2_	1.29, m 1.54, m	34.5
2	CH_2_	1.62, m	29.5	CH_2_	1.62, m	29.5
3	CH	4.08, m	67.4	CH	4.09, m	67.4
4β 4α	CH_2_	1.96, m 1.37, m	33.4	CH_2_	1.95, td (13.8, 3.1) 1.37, br. d (13.8)	33.4
5	CH	2.17, dt (13.0, 2.7)	37.7	CH	2.16, dt (13.8, 3.1)	37.7
6	CH	3.53, m	77.3	CH	3.53, m	77.3
7	CH	4.26, d (2.7)	72.2	CH	4.24, d (2.5)	72.2
8	C		128.0	C		128.1
9	CH	2.24, m	45.5	CH	2.24, m	45.7
10	C		38.6	C		38.7
11β 11α	CH_2_	1.53, m 1.65, m	19.5	CH_2_	1.51, m 1.65, m	19.3
12β 12α	CH_2_	1.83, dt (12.7, 3.7) 1.27, m	37.3	CH_2_	1.78, dt (12.0, 3.4) 1.21, m	36.8
13	C		45.1	C		45.0
14	C		147.4	C		147.0
15β 15α	CH_2_	2.90, ddd (17.1, 9.1, 3.0) 2.36, ddd (17.1, 5.1, 2.1)	34.4	CH_2_	2.89, ddd (17.0, 8.6, 3.2) 2.31, ddd (17.0, 5.7, 2.0)	34.2
16	CH	4.55, td (9.1, 5.1)	79.8	CH	4.57, m	79.7
17	CH	1.45, dd (9.1, 5.6)	62.5	CH	1.46, dd (9.6, 2.8)	62.5
18	CH_3_	0.92, s	20.2	CH_3_	0.89, s	20.6
19	CH_3_	0.83, s	15.4	CH_3_	0.83, s	15.3
20	CH	1.69, m	33.3	CH	2.38, m	37.1
21	CH_3_	1.02, d (6.9)	21.0	CH_3_	1.08, d (7.1)	24.4
22	CH_2_	1.81, m 1.45, m	33.9	CH	5.77, ddd (15.4, 9.2, 1.0)	135.9
23	CH_2_	2.21, m 1.91, m	33.4	CH	5.24, dd (15.4, 8.0)	134.9
24	C		158.3	CH	1.98, m	44.4
25	CH	2.28, dsept (1.0, 6.8)	34.9	CH	1.50, m	34.6
26	CH_3_	1.04, d (6.8)	22.4	CH_3_	0.87, d (6.8)	20.6
27	CH_3_	1.04, d (6.8)	22.6	CH_3_	0.85, d (6.8)	20.2
28	CH_2_	4.74, br. d (1.0) 4.73, br. s	106.7	CH_3_	0.96, d (6.8)	18.3
**GlcNAc**						
1′	CH	4.51, d (8.3)	100.9	CH	4.50, d (8.2)	101.3
2′	CH	3.52, dd (10.4, 8.3)	58.4	CH	3.56, dd (10.4, 8.2)	58.3
3′	CH	3.62, dd (10.4, 8.8)	76.1	CH	3.62, dd (10.4, 8.6)	76.1
4′	CH	3.05, dd (9.8, 8.8)	81.8	CH	3.16, dd (9.7, 8.6)	81.5
5′	CH	3.24, ddd (9.8, 5.4, 2.1)	77.3	CH	3.25, ddd (9.7, 4.5, 2.2)	77.2
6′	CH_2_	3.83, dd (11.5, 2.1) 3.67, dd (11.5, 5.4)	62.9	CH_2_	3.87, dd (11.6, 2.2) 3.74, dd (11.6, 4.5)	62.6
C=O	C		173.9	C		173.8
CH_3_-CO	CH_3_	1.99, s	23.2	CH_3_	1.99, s	23.2
4′-OCH_3_	CH_3_	3.55, s	60.9	CH_3_	3.57, s	60.9

## Supplementary Materials

Copies HRESIMS (Figures S1 and S8), 1H- (Figures S2 and S9), 13C- (Figures S3 and S10), 1H-1H-COSY (Figures S4 and S11), HSQC (Figures S5 and S12), HMBC (Figures S6 and S13), and ROESY (Figures S7 and S14) spectra of compounds 1 and 2, respectively. This material is available free of charge online.
