# Supplementary material for: Two New Steroidal Monoglycosides, Anthenosides A1 and A2, and Revision of the Structure of Known Anthenoside A with Unusual Monosaccharide Residue from the Starfish Anthenea aspera
Source: Molecules. 2018 May 3;23(5):1077. doi: 10.3390/molecules23051077 (PMC6102596; doi:10.3390/molecules23051077)

Supplementary Materials

Two New Steroidal Monoglycosides, Anthenosides A_1 and A_2, and Revision of the Structure of Known Anthenoside A with Unusual Monosaccharide Residue from the Starfish *Anthenea aspera*

Timofey V. Malyarenko[1,2*], Natalia V. Ivanchina[1], Olesya S. Malyarenko[1], Anatoly I. Kalinovsky[1], Pavel S. Dmitrenok[1], Evgeny V. Evtushenko[1], Chau Van Minh[3] and Alla A. Kicha[1]

[1] G.B. Elyakov Pacific Institute of Bioorganic Chemistry, Far Eastern Branch of the Russian Academy of Sciences, Pr. 100-let Vladivostoku 159, 690022 Vladivostok, Russia

[2] Far Eastern Federal University, Sukhanova str. 8, 690000 Vladivostok, Russia

[3] Institute of Marine Biochemistry, Vietnam Academy of Science and Technology, 18 Hoang Quoc Viet, Caugiay, Hanoi, Viet Nam

List

Figure S1. HRESIMS spectrum of anthenoside A_1 (**1**).

Figure S2. ^{1}H-NMR spectrum of anthenoside A_1 (**1**) in CD_3OD.

Figure S3. ^{13}C-NMR spectrum of anthenoside A_1 (**1**) in CD_3OD.

Figure S4. ^{1}H-^{1}H-COSY spectrum of anthenoside A_1 (**1**) in CD_3OD.

Figure S5. HSQC spectrum of anthenoside A_1 (**1**) in CD_3OD.

Figure S6. HMBC spectrum of anthenoside A_1 (**1**) in CD_3OD.

Figure S7. ROESY spectrum of anthenoside A_1 (**1**) in CD_3OD.

Figure S8. HRESIMS spectrum of anthenoside A_2 (**2**).

Figure S9. ^{1}H-NMR spectrum of anthenoside A_2 (**2**) in CD_3OD.

Figure S10. ^{13}C-NMR spectrum of anthenoside A_2 (**2**) in CD_3OD.

Figure S11. ^{1}H-^{1}H-COSY spectrum of anthenoside A_2 (**2**) in CD_3OD.

Figure S12. HSQC spectrum of anthenoside A_2 (**2**) in CD_3OD.

Figure S13. HMBC spectrum of anthenoside A_2 (**2**) in CD_3OD.

Figure S14. ROESY spectrum of anthenoside A_2 (**2**) in CD_3OD.

Figure S1. HRESIMS spectrum of anthenoside A_1 (**1**)

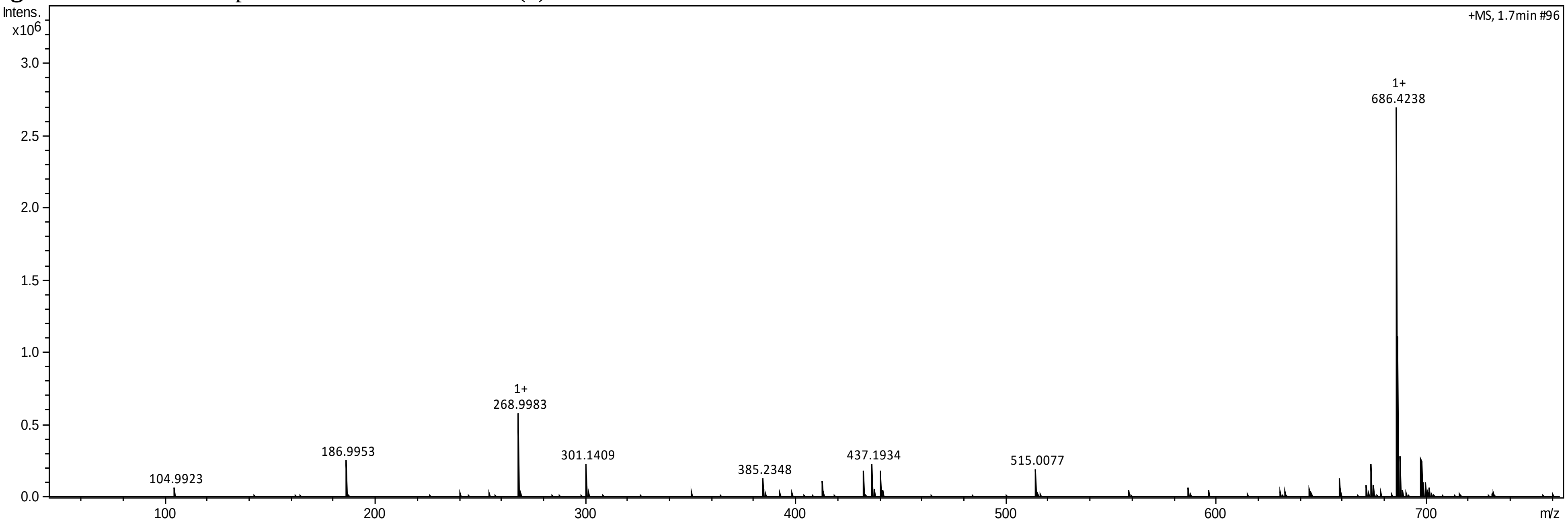

Figure S2. ^{1}H-NMR spectrum of anthenoside A_1 (**1**) in CD_3OD.

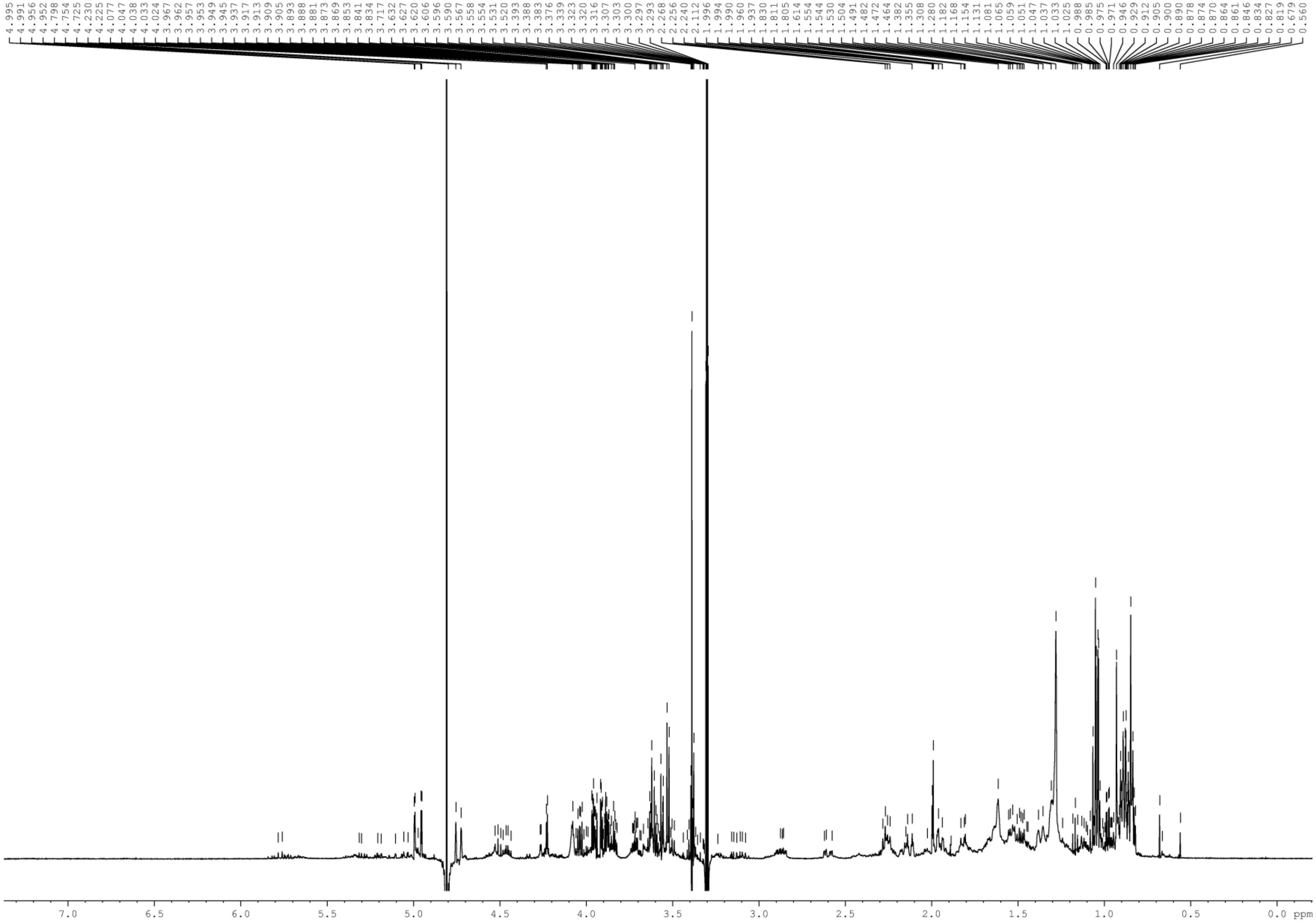

Figure S3. ^{13}C-NMR spectrum of anthenoside A_1 (**1**) in CD_3OD.

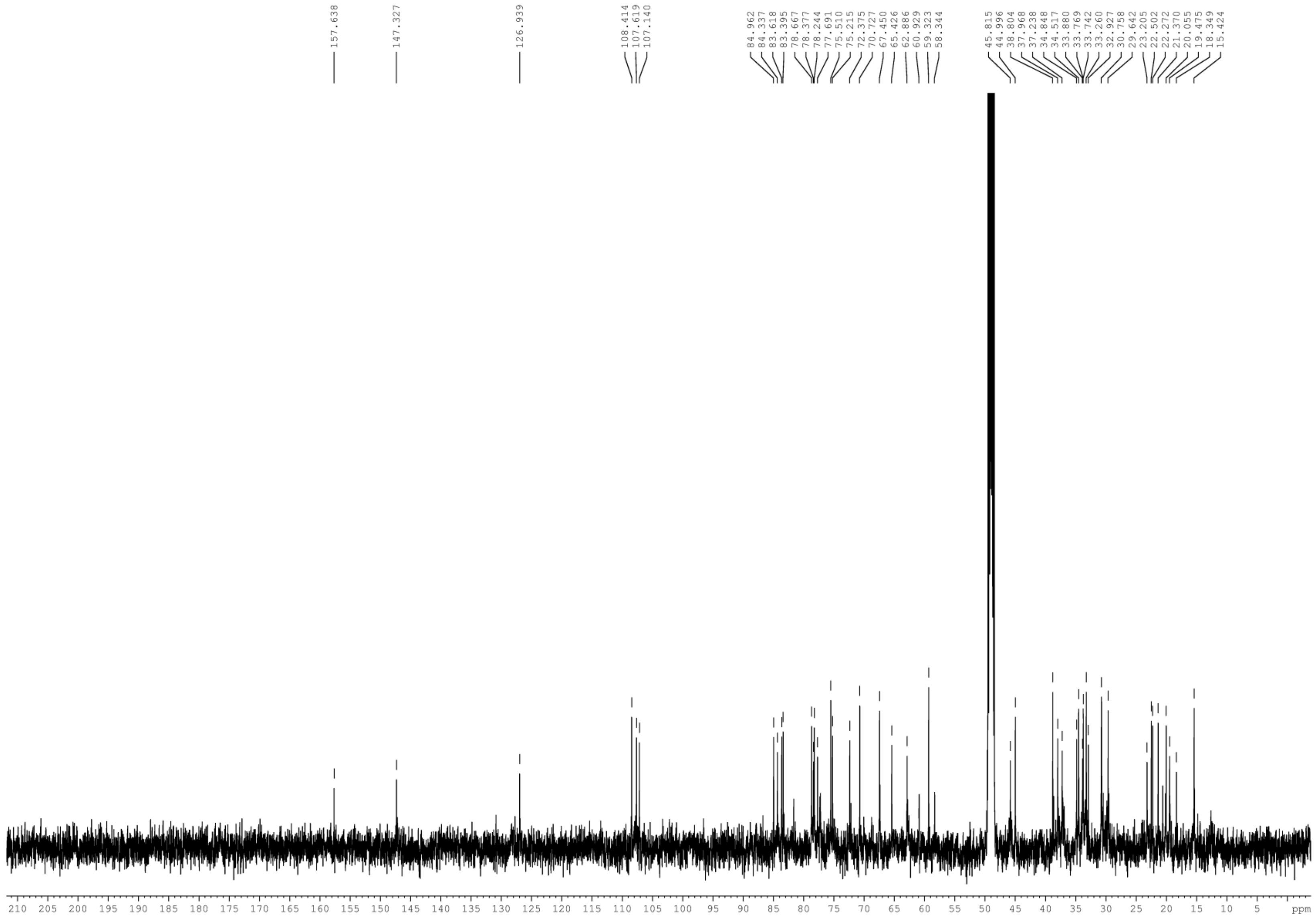

Figure S4. ^{1}H-^{1}H-COSY spectrum of anthenoside A_1 (**1**) in CD_3OD.

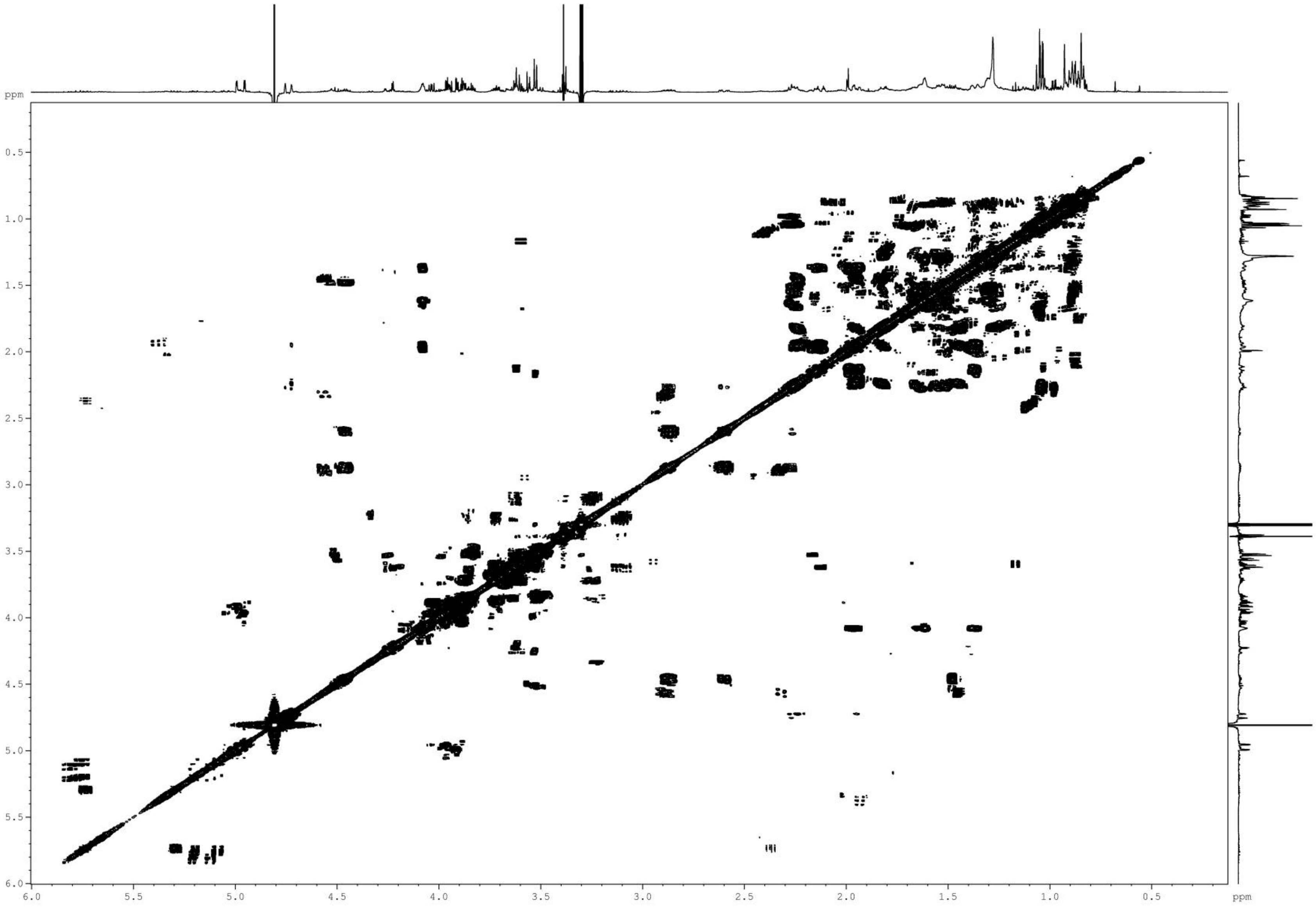

Figure S5. HSQC spectrum of anthenoside A_1 (**1**) in CD_3OD.

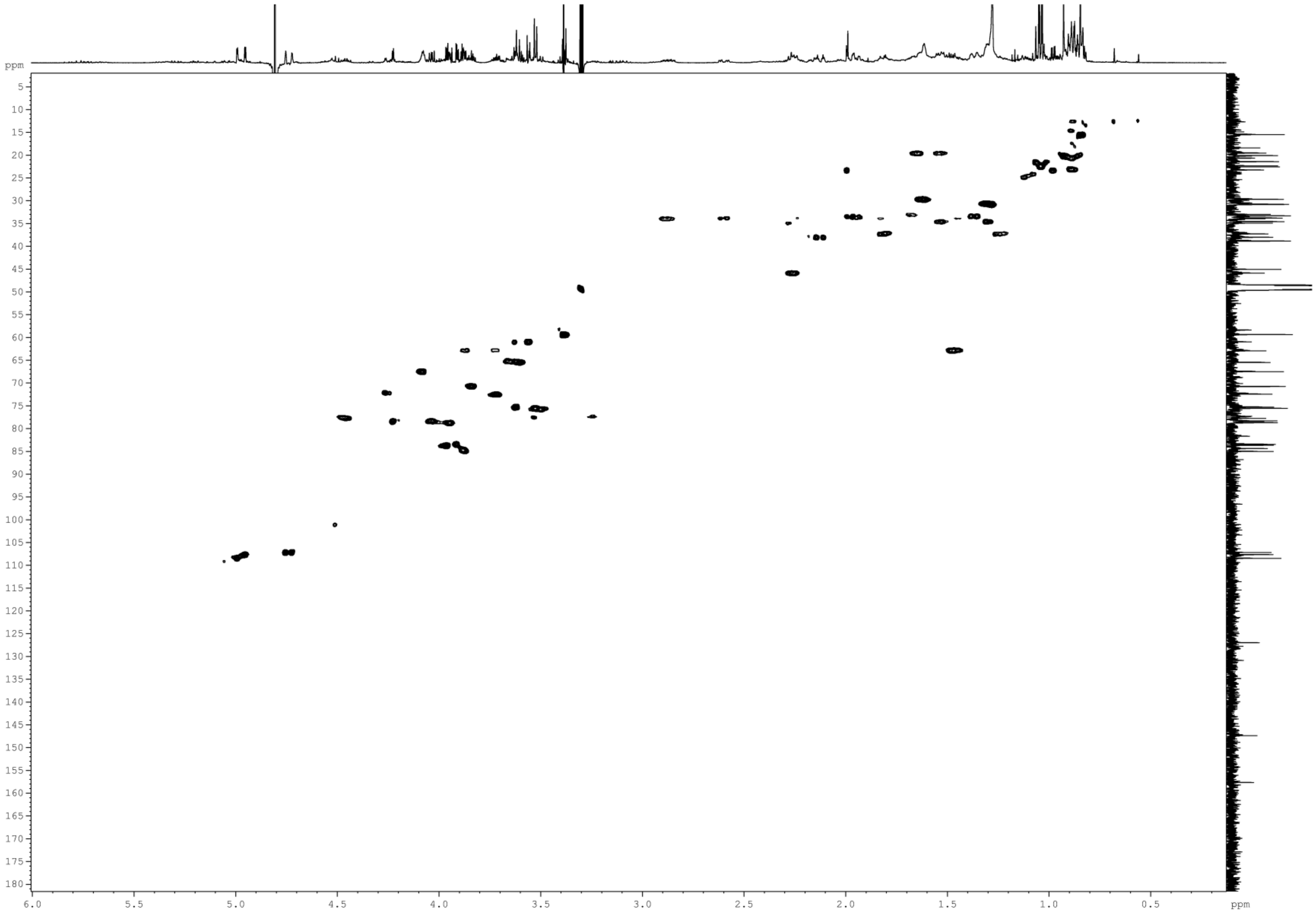

Figure S6. HMBC spectrum of anthenoside A_1 (**1**) in CD_3OD.

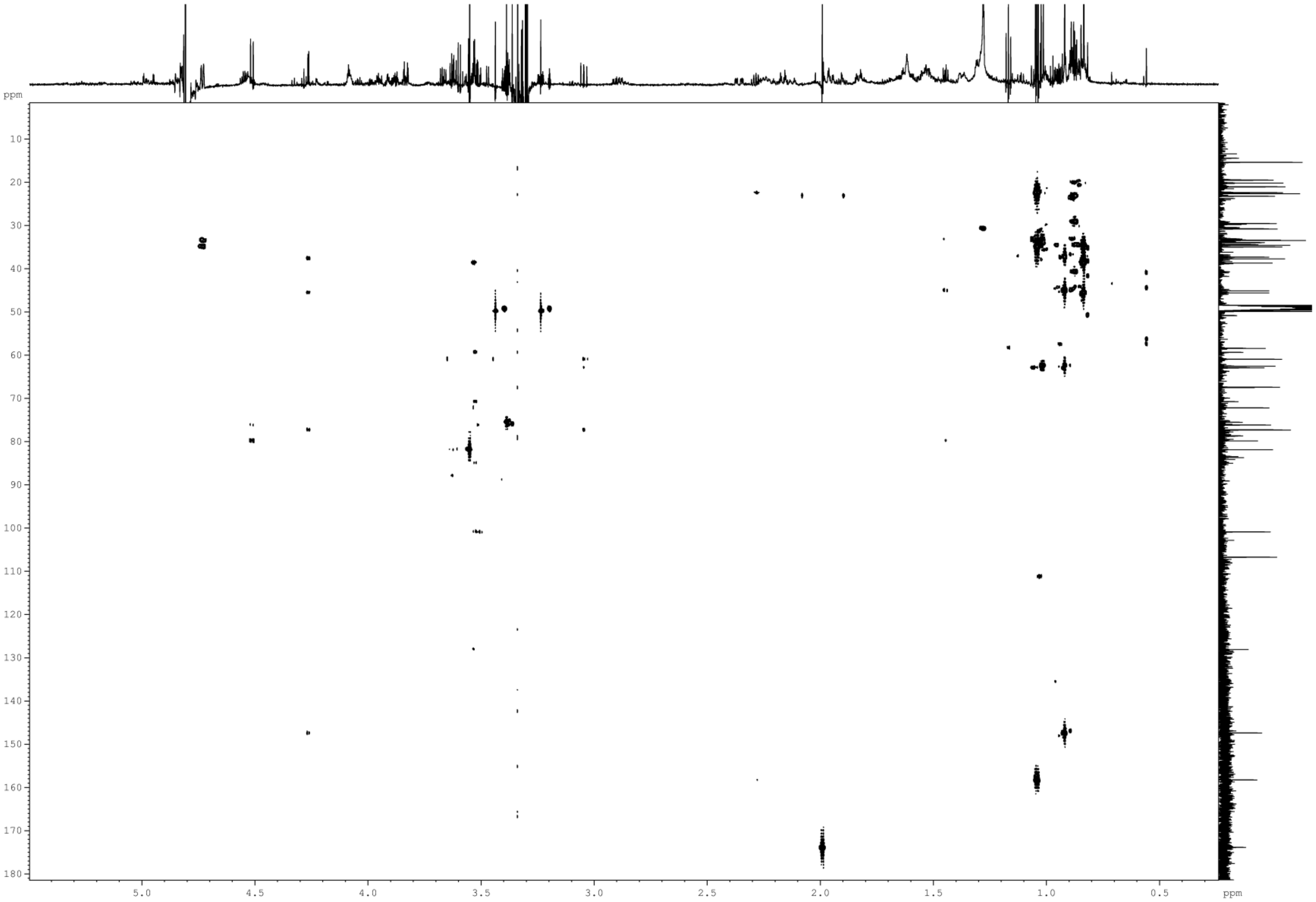

Figure S7. ROESY spectrum of anthenoside A_1 (**1**) in CD_3OD.

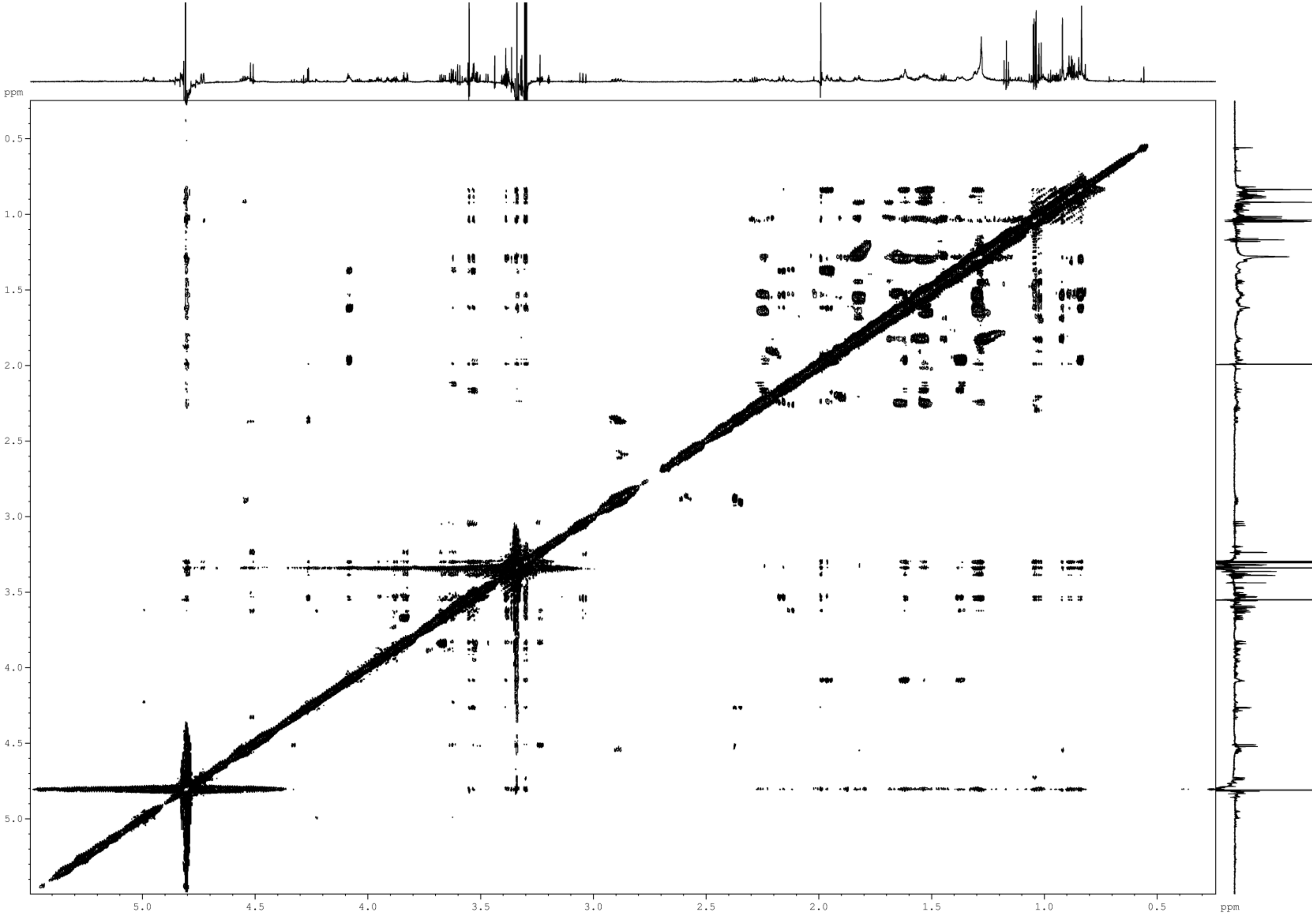

Figure S8. HRESIMS spectrum of anthenoside A_2 (**2**).

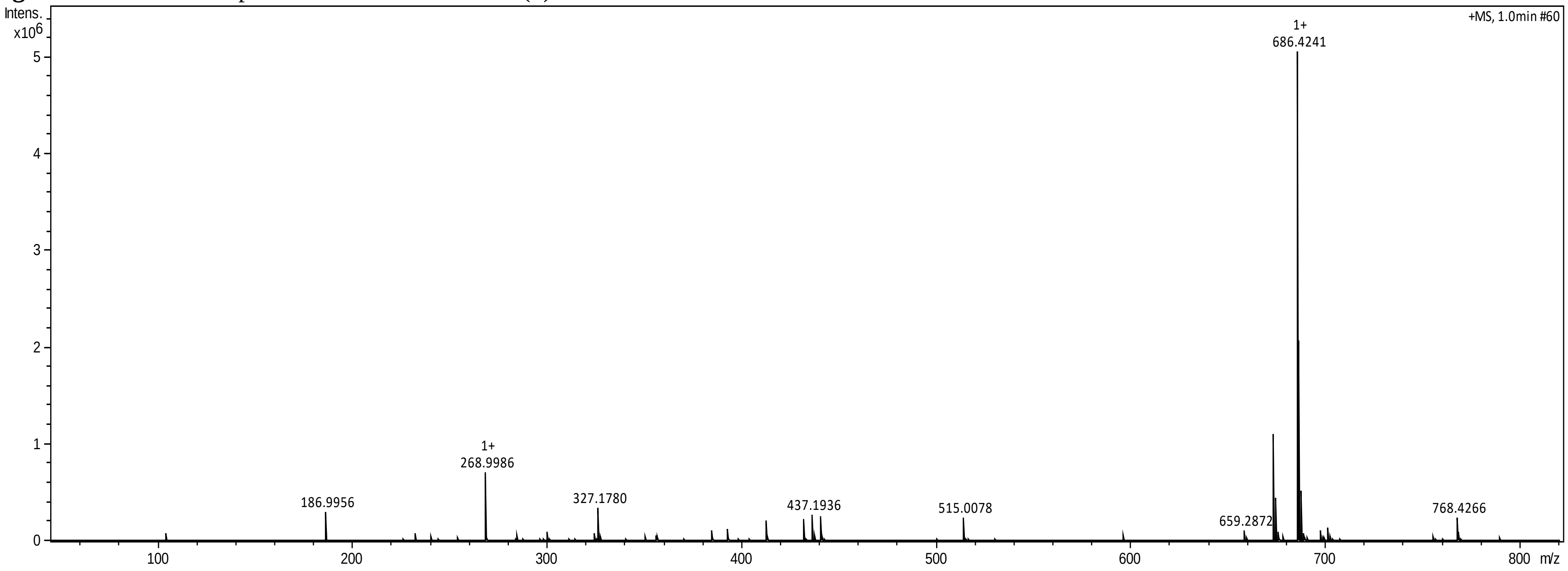

Figure S9. ^{1}H-NMR spectrum of anthenoside A_2 (**2**) in CD_3OD.

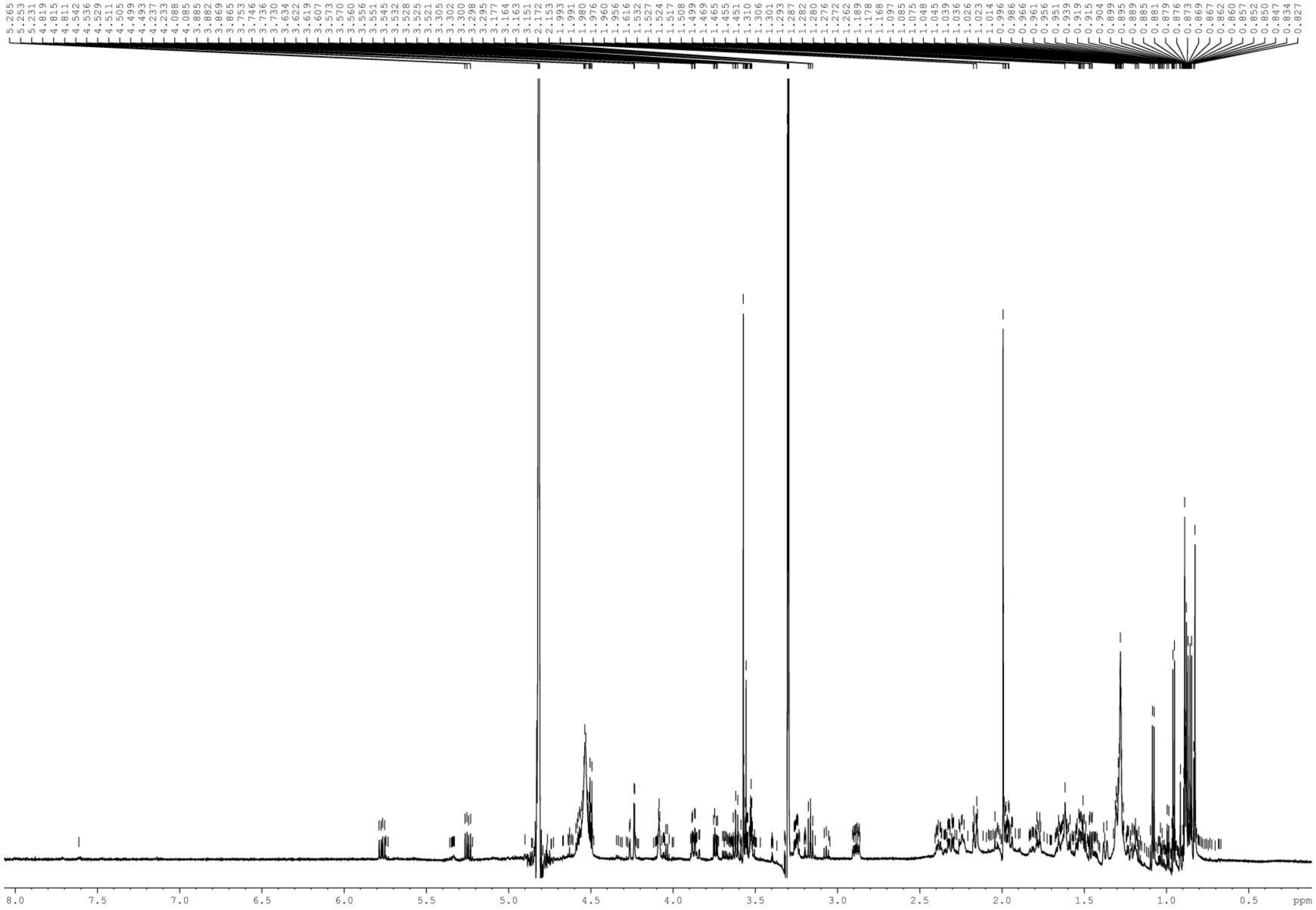

Figure S10. ^{13}C-NMR spectrum of anthenoside A_2 (**2**) in CD_3OD.

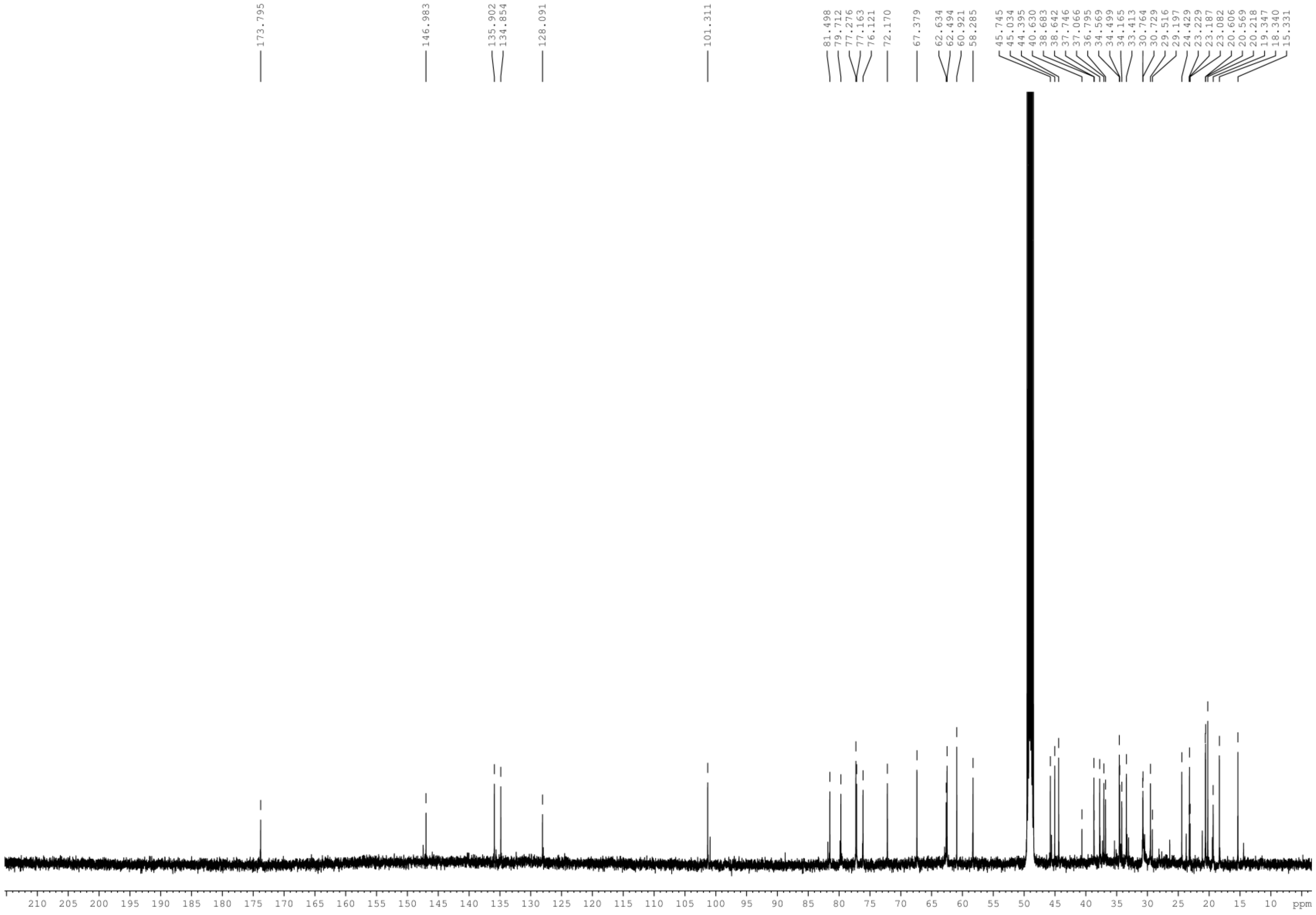

Figure S11. ^{1}H-^{1}H-COSY spectrum of anthenoside A_2 (**2**) in CD_3OD.

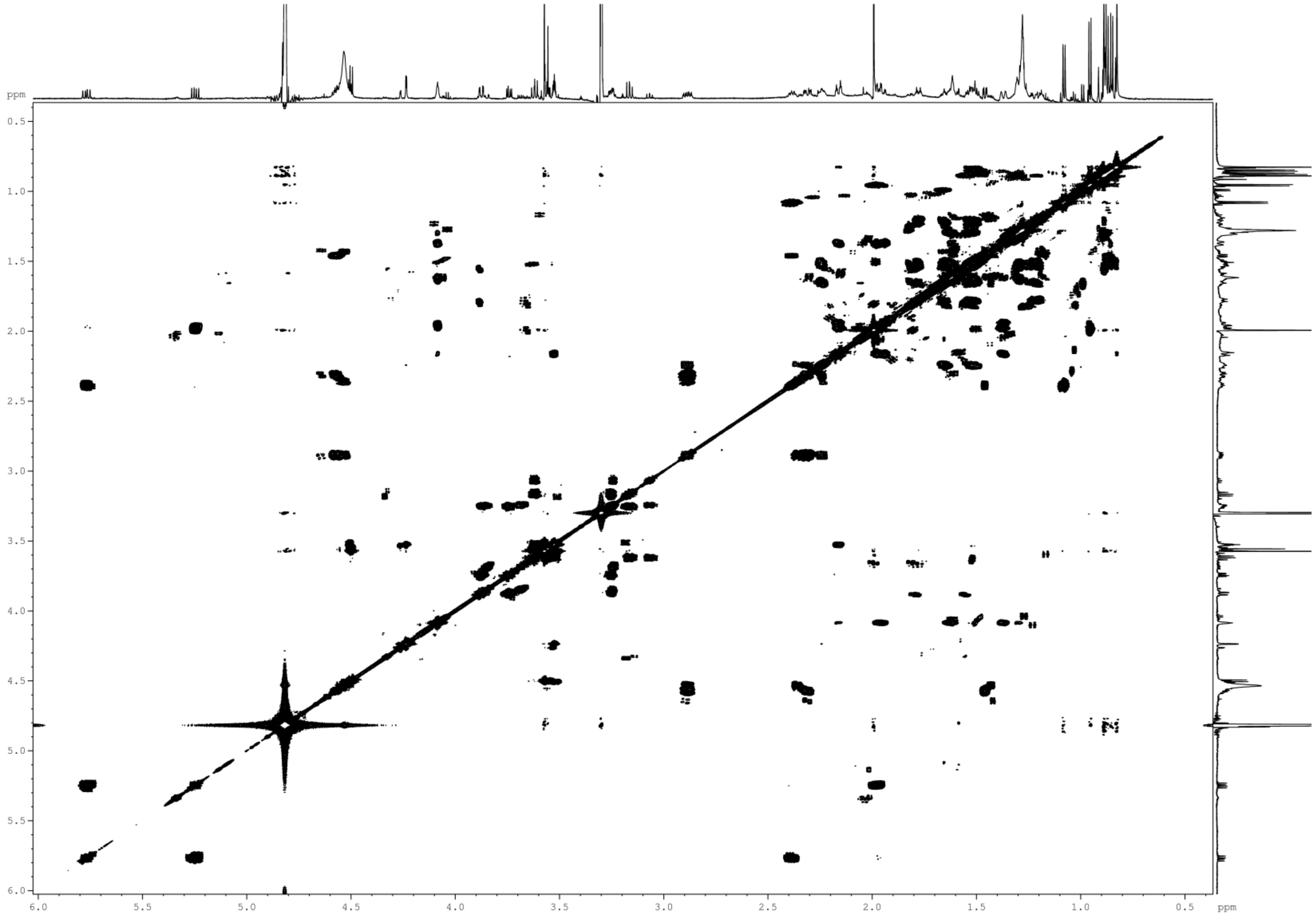

Figure S12. HSQC spectrum of anthenoside A_2 (**2**) in CD_3OD.

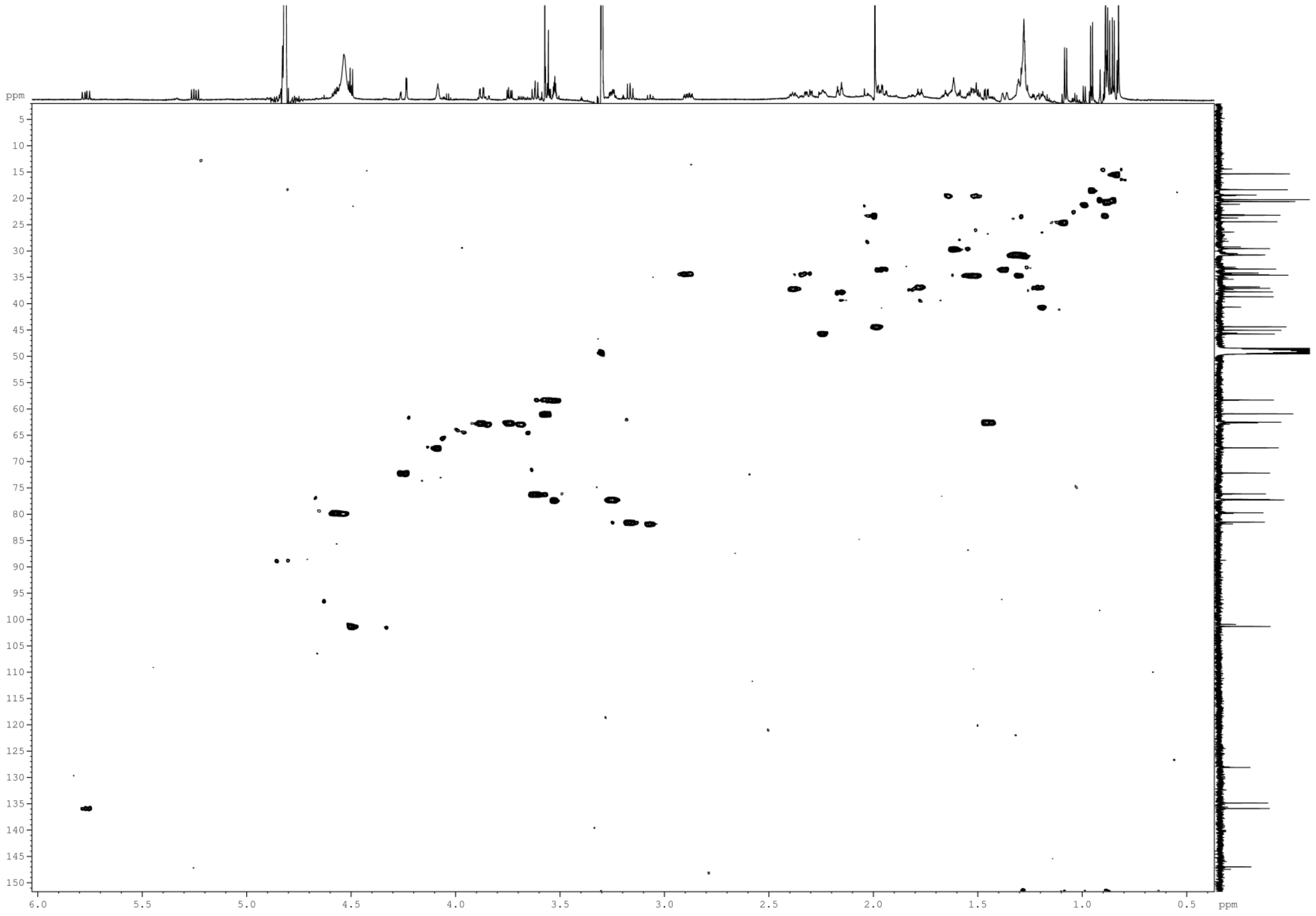

Figure S13. HMBC spectrum of anthenoside A_2 (**2**) in CD_3OD.

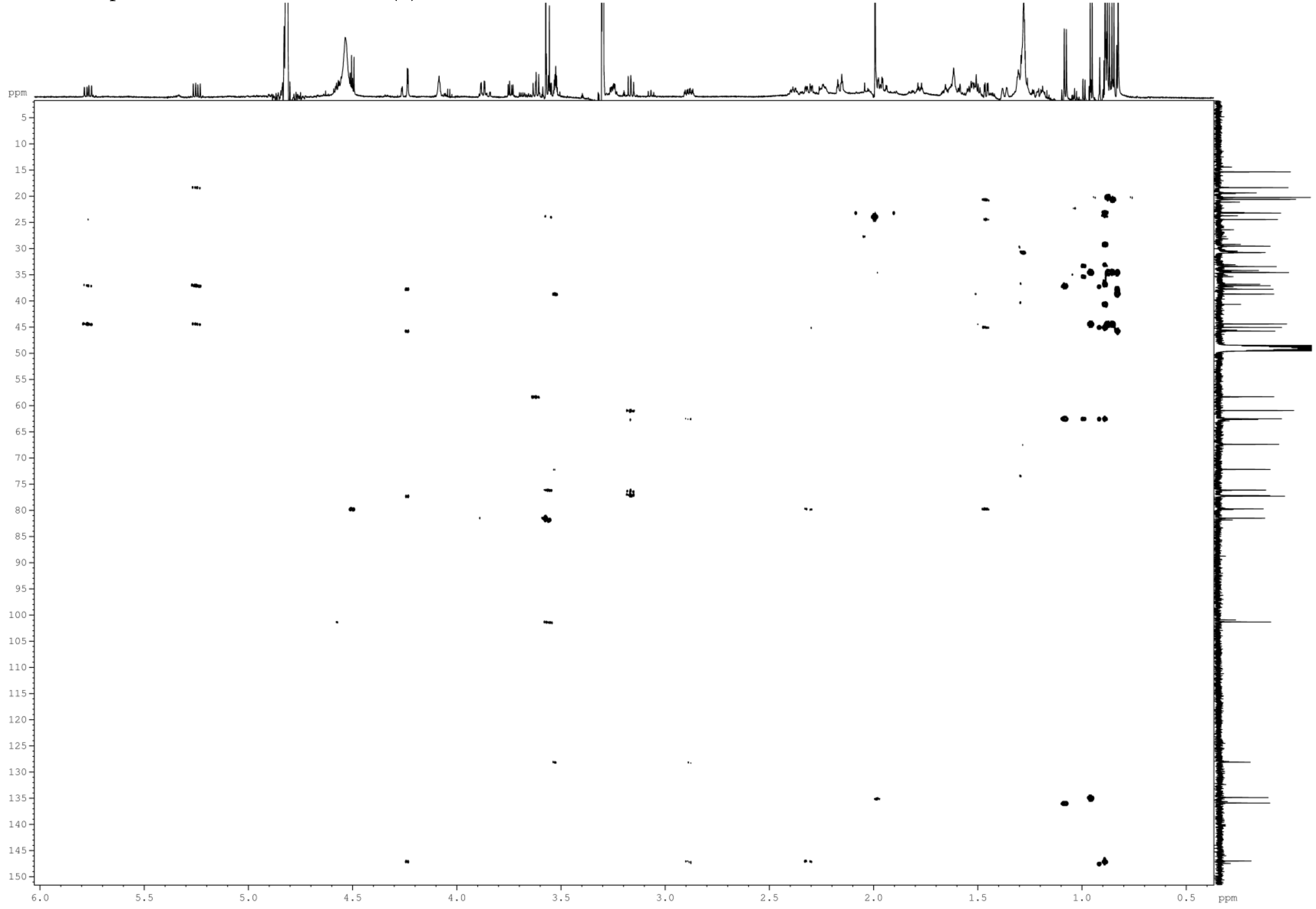

Figure S14. ROESY spectrum of anthenoside A_2 (**2**) in CD_3OD.

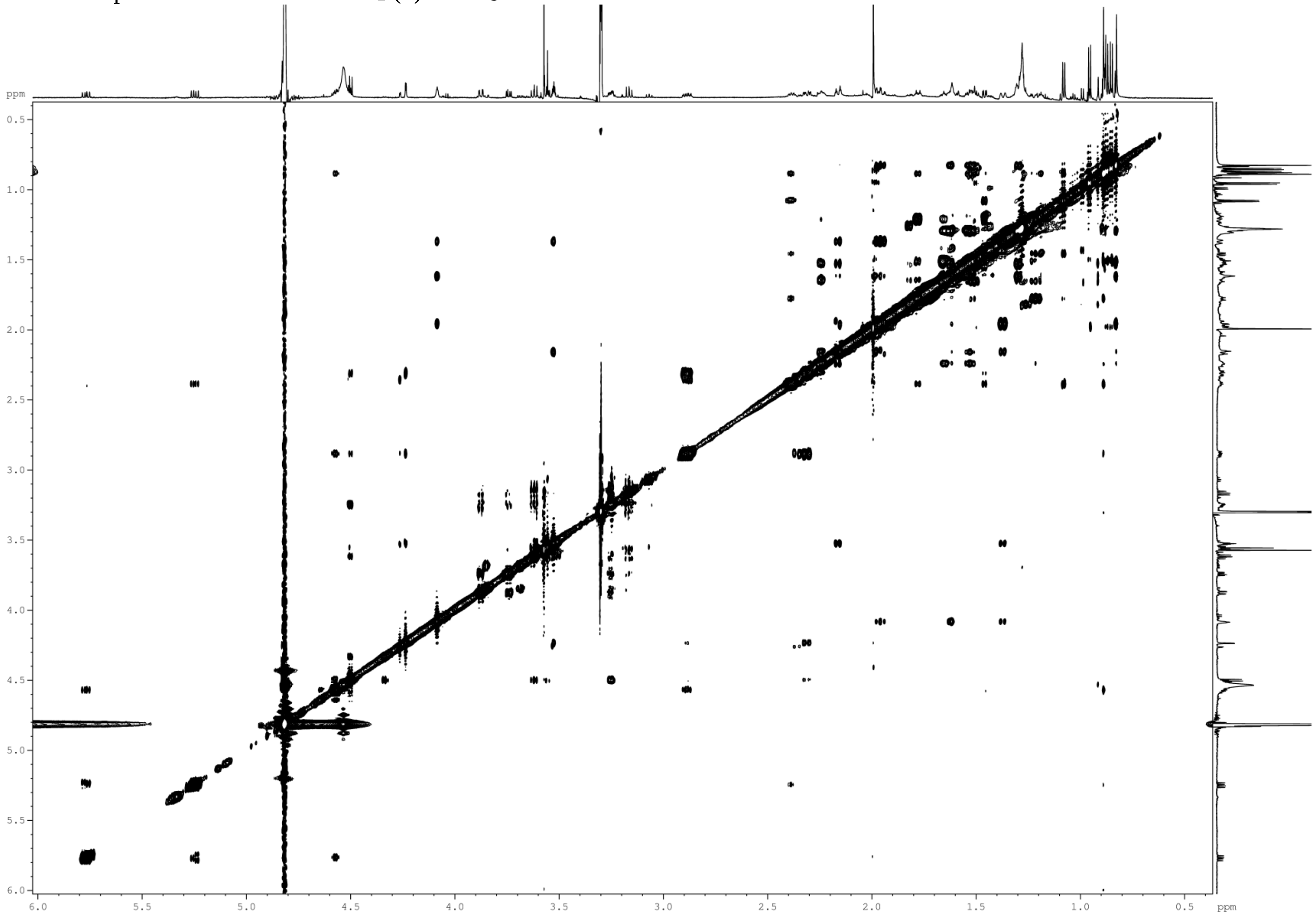

Supplement: Supplementary file 1 [file molecules-23-01077-s001.pdf]
